# The Treatment Perspective of Pediatric Condyle Fractures and Long-Term Outcomes

**DOI:** 10.7759/cureus.30111

**Published:** 2022-10-09

**Authors:** Mehmet Fatih Akkoc, Semra Bulbuloglu

**Affiliations:** 1 Plastic Reconstructive and Aesthetic Surgery, Dicle University Campus, Diyarbakir, TUR; 2 Nursing, Istanbul Aydin University, Istanbul, TUR

**Keywords:** long-term outcome, pediatric dental trauma, treat, denture fracture, mandibular condyle

## Abstract

Purpose: In our study, the characteristics, treatment approach and long-term outcomes of condyle fractures treated in the pediatric plastic surgery and reconstruction unit in the last 10 years were evaluated.

Materials and Methods: This study consisted of two retrospective and prospective sections with the participation of pediatric patients with condylar fractures who were treated in the Plastic, Reconstructive and Aesthetic Surgery clinic of a university hospital in the last 10 years. In the retrospective section, data were obtained from the electronic patient records and patient files regarding the treatment applied, as well as the characteristics of the patient and condyle fractures. In the perspective section, patients were invited to the clinic and the effectiveness of the treatment was evaluated. Statistical analyses were performed with SPSS (Statistical Package for Social Sciences) for IBM 25 package program.

Results: It was determined that 55.8% of the pediatric patients had accompanying facial fractures, and 72.4% had unilateral condyle fractures. It was determined that 59.5% of the pediatric patients underwent intermaxillary fixation (IMF). Physical complications were seen in 6.75% in the long term after treatment.

Conclusion: Falling from height and traffic accidents, which are the most important factors in the occurrence of condyle fractures, should be eliminated by increasing parental attention and awareness. Surgical treatment should be considered in the treatment of pediatric condyle fractures, especially if there are accompanying facial and mandible bone fractures.

## Introduction

Mandibular condyle fractures are usually caused by a direct blow to the face and are less common in children compared to adults [1]. The effectiveness of the treatment protocol is very important in pediatric patients who are in the growth and development period. Because possible complications can damage facial symmetry in the long term. Conservative closed and open interventions used for the treatment of condyle fractures should be decided on the basis of the growth period of the children and should be treated with foresight in this direction. Therefore, the treatment protocol for pediatric condyle fractures is controversial. The prevalence of pediatric facial fractures ranges from 15% to 21% of total oral and maxillofacial fractures [2,3]. Condyle fractures occupy a large area among pediatric mandible fractures [4,5]. There are studies in the literature reporting temporomandibular joint ankylosis or growth disorders [6,7]. In this context, a treatment protocol should be determined to maintain the most appropriate approach for the patient, taking into account the age of the patient and the function of the broken bone.

When planning the treatment and management of maxillofacial fractures, the localization of the fracture, its severity, and accompanying injuries should be considered. For this purpose, anamnesis, maxillofacial region and teeth are examined using an appropriate imaging technique. Maxillofacial fractures are often complex, multiple and asymmetrical. The localization, extent and displacement of the maxillofacial fracture are of great importance [8,9]. After the necessary determinations are made, conservative, closed reduction, intermaxillary fixation (IMF) and/or open reduction internal fixation (ORIF) are the treatment protocols that are frequently used in maxillofacial fractures [10]. Monitoring the results is as important as an effective and safe treatment protocol in pediatric condyle fractures. Studies in which the results of the treatment are evaluated are very important in the process of evaluating the compliance of the treatment applied by the physician with the expected results, maintaining the correct practices, and developing/changing the inadequate techniques. In this study, we examined in detail the characteristics, treatment approach and long-term outcomes of condyle fractures treated in the pediatric plastic surgery and reconstruction unit in the last 10 years.

## Materials and methods

Our study was carried out as a descriptive and cross-sectional two-stage (retrospective and prospective) study. Pediatric patients with condyle fractures who were treated at the Plastic and Aesthetic Surgery Clinic of Diyarbakır Dicle University Medical Faculty Hospital between June 2010 and June 2020 were included in the sample. The purposive sampling method was used in sample selection. All pediatric condic fracture patients treated within the specified date range were included in the sample. Patient data were reviewed retrospectively using historical computer records, patient files, and electronic records and recorded in a data form developed by the researchers. The sociodemographic characteristics of the patients, the etiology of condyle fracture, the intervention applied, and the medical and surgical treatment information were recorded. Pediatric patients who had condyle fractures in the prospective department and were treated by the hospital where the study was conducted were invited to the hospital by appointment system and were examined one by one. Long-term outcomes and complications were determined according to the patient's history, clinical examination findings and radiographic imaging methods.

Statistical analyzes of the results obtained in the study were performed with Statistical Package for Social Sciences (SPSS) for IBM 25 package program. Descriptive statistical methods were used in data evaluation. ANOVA was performed to determine the statistical significance of the differences between the variables. Regression analyses were used. The results were evaluated at a 95% confidence interval and p<0.05 significance level.

This research has been approved by Dicle University Medical Faculty, Department of Plastic, Reconstructive and Aesthetic Surgery Institutional Review Board (IRB). Before starting the study, the necessary permissions were obtained from Dicle University Research and Training Hospital’s Clinical Research Ethics Committee (Date: January 21, 2022, Number: 14). In compliance with the principles of the Declaration of Helsinki, the participants and the parents of participants under the age of 18 were informed about the study, and the researcher read them the Informed Consent Form. Voluntarily agreed to participate were included in the study after they provided verbal and written informed consent.

## Results

The introductory characteristics of patients with condylar fractures and information on the treatment process are given in Table 1. All patients were accessed. According to the table, 62.6% of the pediatric patients were in the 0-3 age range, and 73.6% were male. Considering the etiology of the fracture, it was determined that 59.5% were caused by falling from a height and 25.8% were caused by traffic accidents. It was determined that 55.8% of the pediatric patients had accompanying facial fractures, and 72.4% had unilateral condyle fractures. It was determined that 59.5% of the pediatric patients underwent IMF. In addition to condyle fractures, the rate of patients with more than 2 accompanying fractures was 9.8%, and the rate of accompanying mandible fractures was 42.3%. When the long-term outcomes after the treatment were examined, it was found that physical complications were 6.75%, the rate of pediatric patients with slowed growth and development was 25.8%, and the development of malnutrition was 19%. It was determined that the rate of children who were diagnosed with a psychiatric diagnosis after condylar fracture treatment was 12.5%.

**Table 1:** Information on introductory characteristics and treatment process of patients with condylar fractures (n=163) *All of the intra and extracapsular fractures in our study accompany unilateral and bilateral fractures.

Introductory Features	n	%
Age Groups		
0-3 Ages	102	62.6
4-7 Ages	31	19
Ages 8 and up	30	18.4
Gender		
Male	120	73.6
Female	43	26.4
Fracture Etiology		
Traffic accidents	42	25.8
Being Battered	15	9.3
Falling from high	97	59.5
Other (Sports injuries, Home accidents etc.)	9	5.4
Presence of Concomitant Facial Fracture		
Yes	91	55.8
No	72	44.2
Condyle Fracture Type		
Unilateral	118	72.4
Bilateral	8	4.9
Other* Intra-extracapsular)	37	22.7
Treatment Applied		
Conservative	20	12.3
Intermaxillary Fixation (IMF)	97	59.5
Open Reduction Internal Fixation (ORIF)	8	4.9
ORIF+IMF	38	23.3
Associated Facial Fractures		
Zygomatic Arch	4	2.5
Zygoma	8	4.9
Orbit	1	0.6
Nasoorbitoethmoid	2	1.2
Maxilla	5	3.1
Nasal	3	1.8
Dentoalveolar	12	7.4
Number of Associated Facial Fractures		
Fracture at 1 area	14	8.6
Fracture at 2 areas	5	3.1
Fracture at more than 2 areas	16	9.8
Associated Mandible Fractures*		
Parasymphysis	17	10.4
Coronoid	3	1.8
Ramus	6	3.7
Without symphysis	32	19.6
Corpus	13	8
Angulus	2	1.2
Total	69	42.3
Long-Term Outcomes After Treatment		
Development of physical complications after at least 6 months Growth and development slowed down	11	6.75
Malnutrition development	42	25.8
School success decreased after condyle fracture	31	19
Any psychiatric diagnosis after condyle fracture (anxiety, depression, bedwetting, etc.)	5	3
Long-Term Outcomes After Treatment	20	12.5

The predictors of the treatment method applied in condyle fractures are given in Table 2. According to the table, the type of facial fractures, mandible fractures, and condyle fractures are predictors of the treatment method applied, and this result was found to be statistically significant (p=0.000).

**Table 2:** Predictors of treatment method applied in condyle fractures (n=163) ^a^Dependent Variable: Applied treatment method ^b^Predictors: (Constant), Facial fractures (zygomatic arch, zygoma, orbita, nasoorbitoethmoid, maxilla, nasal, dentoalveolar fractures), Mandible Fractures (parasymphysis, coronoid, ramus, symphysis, corpus, angulus fractures), Condyle fracture type *p<0.01

ANOVA^a^						
Model		Sum of Squares	df	Mean Square	F	Sig.
1	Regression	15.319	2	7.659	37.945	.000^b*^
	Residual	3.633	18	.202		
	Total	18.952	20			

**Figure 1:**
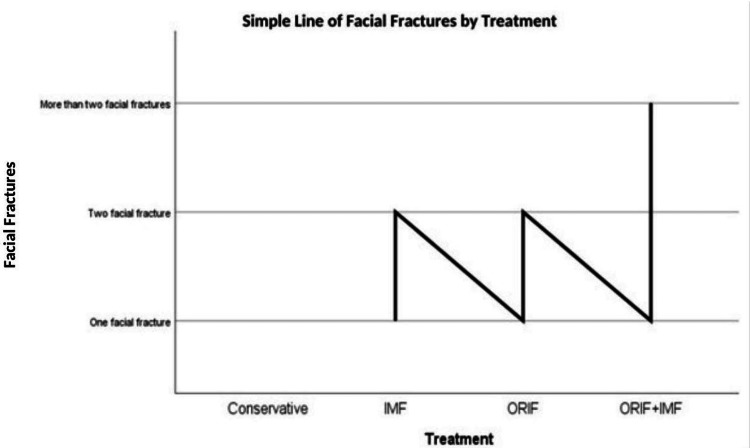
Treatment method applied to condyle fractures according to the presence of associated facial fractures (n=163) Facial fractures include zygomatic arch, zygoma, orbit, nasoorbitoethmoid, maxilla, nasal, dentoalveolar fractures. IMF: Intermaxillary Fixation, ORIF: Open Reduction Internal Fixation

In Figure 1, the treatment method applied to condyle fractures according to the presence of accompanying facial fractures is schematized. According to the figure, it was determined that a conservative treatment method was preferred in the repair of condyle fractures in the absence of accompanying facial fractures, and ORIF or ORIF+IMF was preferred in the presence of 1 or more facial fractures.

**Figure 2:**
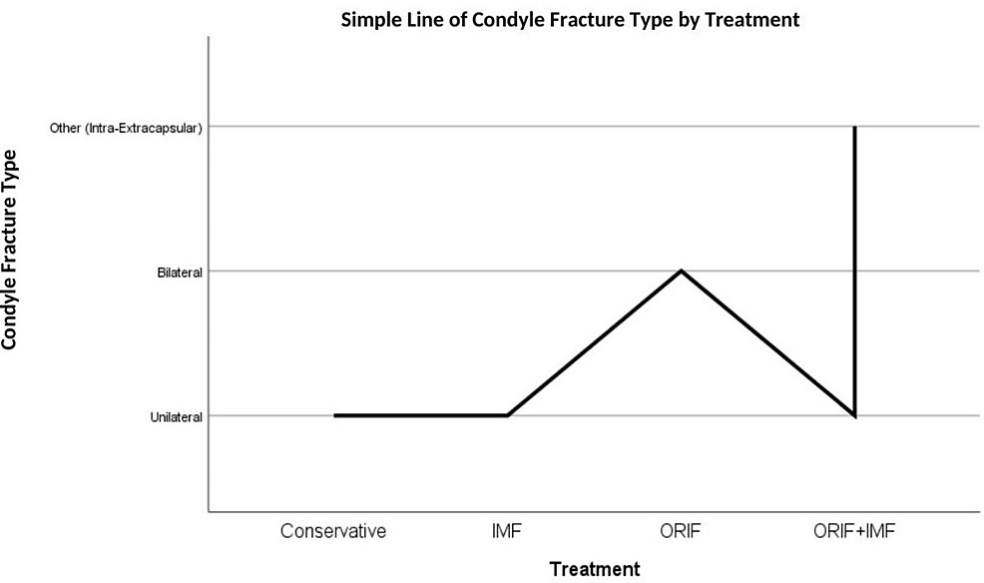
Treatment method according to condylar fracture type (n=163) All of the intra and extracapsular fractures in our study accompany unilateral and bilateral fractures. IMF: Intermaxillary Fixation, ORIF: Open Reduction Internal Fixation

In Figure 2, the treatment method applied according to the condyle fracture type is schematized. In the figure, it was determined that conservative, IMF, ORIF, and ORIF+IMF were preferred in the treatment of unilateral condyle fractures. Furthermore, it was seen that IMF, ORIF, and ORIF+IMF are applied in the repair of bilateral condyle fractures and ORIF+IMF is preferred in the presence of intra-extra capsular fractures. 

Table 3 shows the regression analysis between the treatment method applied and the condyle fracture types and facial fractures. Therefore, a statistically significant correlation was found between the treatment method applied and the type of condyle fracture and the type of mandible fracture (p=0.000). Although not statistically significant, a negative correlation was found between facial fractures and the treatment method (p=.770).

**Table 3:** Advanced physical complications and management at least six months after condylar fracture treatment (n=163)

Complications	n	%	Fracture and treatment information	Conclusion
Malocclusion	3	1.8	In addition to condyle fracture, ARIF+IMF was applied with facial fracture in 3 areas.	He was referred to the physical therapy clinic.
Deviation in opening mouth	1	0.6	There were intracapsular and bilateral condyle fractures, ARIF+IMF was applied	He was referred to the physical therapy clinic.
Ankylosis	2	1.2	One had intracapsular and bilateral condyle fractures, no accompanying facial trauma and IMF applied	He was referred to the physical therapy clinic.
Limitation in jaw movements	2	1.2	The other had intracapsular and bilateral condyle fractures, concomitant ramus fracture, ARIF+IMF was applied	He was referred to the physical therapy clinic.
Asymmetry	1	0.6	ARIF+IMF applied after bilateral condyle fracture	He was referred to the physical therapy clinic.
Chronic pain	2	1.2	In addition to condyle fracture, ARIF+IMF was applied with facial fracture in 3 areas.	He was referred to the algology clinic.

Complications developed at least six months after condyle fracture treatment and their management are listed in Table 4. According to the table, malocclusion developed in 1.8% of pediatric patients, deviation in mouth opening in 0.6%, ankylosis in 1.2% and limitation in jaw movements at the same rate, asymmetry in 0.6% and chronic pain in 1.2%.

**Table 4:** Regression analysis between the treatment method applied and condylar fracture types and facial fractures (n=163) ^a^Dependent Variable: Applied treatment method *p<0.01

Coefficients^a^						
Model		Unstandardized Coefficients		Standardized Coefficients	t	p
		B	Std. Error	Beta		
1	(Constant)	2.310	.262		8.826	.000*
	Condyle fracture type	.921	.114	.912	8.079	.000*
	Facial fractures	-.048	.163	-.034	-.297	.770
	Mandible fractures	1.000	.045	.940	22.292	.000*

## Discussion

Condyle fractures have an important place among mandible fractures. Condyle, mandible and facial fractures are most common in pediatric patients due to falls and traffic accidents [11-13]. In our study, falling from a height was the first in the etiology of condylar fractures, and traffic accidents were in second place. In one study, it was noted that 240 pediatric facial fractures were mostly seen in the 11-14 age group with a rate of 27.5%. In the same study, it was reported that complications developed in 18.33% of pediatric patients after surgery, and the most common complications were deviation in mouth opening and growth disorder [11]. In our study, the treatment methods applied to pediatric patients with a condylar fracture who applied to a training and practice hospital in the last 10 years and the long-term outcomes of these treatments were examined. All pediatric patients who had condyle fractures during this period were in the 0-7 age range. In our study, it was determined that the rate of pediatric patients who developed physical complications after condyle fracture repair was 6.75%.

Although conservative treatment is mostly recommended in the treatment of pediatric condyle fractures [14-16], it was found that traumatized condyle is involved in the etiology of facial asymmetry in young adulthood [17]. In our study, the rate of patients who received conservative treatment was 12.3%. None of the condyle fractures that were treated conservatively were accompanied by other facial and mandible fractures. Moreover, ORIF+IMF was preferred in the presence of an intra-extra capsular fracture. Care should be taken in the selection of conservative treatment in the repair of condyle fractures. Because individuals who were not treated in childhood may apply to the hospital for surgical treatment in order to obtain an aesthetic appearance in later years. In addition to aesthetic problems, when condylar fractures are not treated surgically in childhood, they may cause pain and crepitation during chewing due to ruptured or displaced discs in advanced stages [18]. In the literature review, it was found that in the treatment of pediatric facial fractures, not only the bone but also the intra-articular soft tissues are seriously injured in all fractures and dislocations, the glenoid fossa protrudes with the medial fracture in condyle dislocations, and the posterior ligament is often torn [16]. For this, it is of great importance to revise the intra-articular soft tissues in all condyle dislocations and to fix the disc in place by reducing it [18]. In our study, 59.5% of pediatric condyle fractures were treated with IMF, and in the presence of 1 or more facial fractures, IMF, ORIF or ORIF+IMF were preferred. In our study, it was seen that IMF, ORIF, ORIF+IMF are applied in the repair of bilateral condyle fractures and ORIF+IMF is preferred in the presence of intra-extra capsular fractures. While determining the treatment protocol for condyle fractures, it is still not possible to predict which methods will be determined very clearly. As a matter of fact, no statistically significant relationship was found between facial fractures and the treatment method in our study (p=0.770). The surgeon's observation, past experience and patient-related factors are determinants of the treatment method to be applied. In our study, it was concluded that the type of condyle fractures and mandibular fractures are predictors for the treatment method applied (p=0.000).

In this study, the rate of pediatric patients whose growth and development slowed down after condyle fracture was determined as 25.8% and malnutrition development as 19%. It is thought that surgical treatment of pediatric condyle fractures prevents growth and development [15]. It is also argued that the surgical treatment of condyle fractures has nothing to do with growth disorder [17]. The usage of low-volume and high-calorie enteral nutrition products after a condyle fracture can help meet the protein and energy deficit of pediatric patients who are in the growth and development period, and since the products are in liquid form, the consumption of condylar patients is easy. Most of the time, pediatric patients suffer from malnutrition in the advanced stages because they cannot be fed according to their needs. Increased catabolism and high protein deficit due to surgery cause nutritional disorders.

This study, it was found that malocclusion developed in 1.8% of pediatric patients after condylar fracture, deviation in opening mouth in 0.6%, ankylosis in 1.2% and limitation in jaw movements at the same rate, asymmetry in 0.6% and chronic pain in 1.2%. Intracapsular fractures have a higher risk of disc displacement and condylar neck fracture [19-21]. In the literature, it was reported that the most common complications and their rates are ankylosis 0.8% and limitation of jaw movement 3.92%, deviation in mouth opening 5.4%, malocclusion 0.8% [22], and facial nerve injury 8.6% [23]. Complications that develop after condylar fracture treatment are affected by parameters such as the surgeon's skill and experience, choosing the appropriate treatment option, post-operative care, and physical therapy support. Therefore, complication development rates also differ. The fact that our study was conducted in a single center was accepted as a principle of limitation. The findings of this study cannot be generalized to the world population as it reflects the dynamics of its sample group. Since the age groups of the patients and the reason for the occurrence of condylar fractures vary, and the treatment protocol is determined according to patient-related factors, the lack of universal validity of the treatment methods used in our study is among the other limitations.

## Conclusions

In pediatric condyle fractures, the appropriate treatment option should be determined by considering the presence of accompanying fracture, the degree of the existing fracture, and patient-related parameters. Complications can be fatal if the most appropriate treatment method is not applied to the patient, and it can cause a significant burden and responsibility in the patient's life. It is very important to prevent condyle fractures first, and parental attention and awareness should be strengthened in this direction. Surgical treatment should not be avoided in the presence of other accompanying facial and mandibular fractures for pediatric patients with condyle fractures. In the postoperative period, the patient should be well-fed, and psychological support should be provided. Awareness regarding post-operative care should be developed. Condyle fracture incorrect repair should be performed with appropriate techniques by experienced surgeons as it can cause significant complications. Condyle fractures that are not repaired with appropriate techniques may require surgical intervention at advanced ages, may impair facial aesthetics and may experience problems in speech and facial expressions. Therefore, surgical treatment can be considered one of the most effective and safe treatment methods in condyle fracture repair.
